# Chinese Herbal Preparation SaiLuoTong Alleviates Brain Ischemia *via* Nrf2 Antioxidation Pathway–Dependent Cerebral Microvascular Protection

**DOI:** 10.3389/fphar.2021.748568

**Published:** 2021-11-02

**Authors:** Xiao-Di Fan, Ming-Jiang Yao, Bin Yang, Xiao Han, Ye-Hao Zhang, Guang-Rui Wang, Peng Li, Li Xu, Jian-Xun Liu

**Affiliations:** ^1^ Institute of Basic Medical Sciences, Xiyuan Hospital of China Academy of Chinese Medical Sciences, Beijing, China; ^2^ Key Laboratory of Pharmacology of Chinese Materia Medica, Beijing, China; ^3^ The Department of Pathology, Xiyuan Hospital of China Academy of Chinese Medical Sciences, Beijing, China

**Keywords:** cerebral ischemia, SaiLuoTong capsule, brain microvascular endothelial cells, anti-oxidation, nuclear factor–E2–related factor 2

## Abstract

Stroke is one of the most devastating diseases worldwide. The Chinese herbal preparation SaiLuoTong (SLT) capsule showed outstanding therapeutic effects on stroke and its sequelae. The aim of this study was to further elucidate its therapeutic mechanism. We duplicated a permanent cerebral ischemia model in rats by MCAO and used SLT (33 and 16.5 mg/kg) to intervene. The results showed SLT dose dependently decreased infarction volumes, relieved neuron degeneration and loss, and ameliorated neurological functions, and the dose of 33 mg/kg had statistical significance (compared with the model group, *p* < 0.05); SLT of 33 mg/kg also significantly inhibited the elevation in brain water content and the loss in claudin-1 and occludin expressions; additionally, it significantly increased nucleus translocation of Nrf2, elevated the expression of HO-1, and raised the activity of SOD and content of GSH (compared with the model group, *p* < 0.05 or 0.01). These results testified SLT’s anti-brain ischemia effect and hint this effect may be related to the protection of brain microvascular endothelial cells (BMECs) that is dependent on the Nrf2 pathway. To further testify, we cultured hCMEC/D3 cells, duplicated OGD/R model to simulate ischemia, and used SLT (3.125, 6.25, and 12.5 mg/L) to treat. SLT dose dependently and significantly inhibited the drop in cell viabilities, and activated the Nrf2 pathway by facilitating Nrf2 nucleus translocation, and increasing HO-1 expression, SOD activity, and GSH content (compared with the model group, *p* < 0.05 or 0.01); last, the anti-OGD/R effects of SLT, including raising cell viabilities, inhibiting the elevation in dextran permeability, and preserving expressions of claudin-1 and occludin, were all abolished by Nrf2 siRNA interference. The *in vitro* experiment undoubtedly confirmed the direct protective effect of SLT on BMECs and the obligatory role of the Nrf2 pathway in it. Collectively, data of this study suggest that SLT’s therapeutic effect on brain ischemia is related to its Nrf2-dependent BMECs protection.

## Introduction

Stroke is one of the leading causes of disability and death worldwide, which produces immense health and economic burdens. Taking the case of China, tens of millions of people suffer from stroke each year, in which nearly ten percent of them die, and most of the remaining people are afflicted to different extents by the sequelae such as sensory and motor impediments, cognition impairments, and affective and speech disorders, which badly influences their lives as well as their families.

Most of strokes are the ischemic type, which occupies 80% of the total cases. It is usually triggered by obstructions of one or more cerebral arteries, which then lead to a critical reduction of regional cerebral blood flow, causing a waterfall-like cascade, and finally resulting in massive neuron deaths (Donnan et al., 2008). In this damage cascade, the ruin of the brain–blood barrier (BBB) plays a pivotal role.

The endothelium of cerebral microvascular is unique compared to that in other tissues, as they have continuous intercellular tight junctions (TJs) and efflux transporters, and thus the endothelium and their TJs form a barrier-like structure, which can greatly limit both the paracellular and transcellular diffusion of vascular inclusions, and constitute the BBB (Bazzoni et al., 2004; Abbott et al., 2006).

Growing evidences demonstrate that brain ischemia causes loss of endothelial cells and TJs, leading to enhanced BBB permeability including not only extravasations of blood plasma constituents and some neurotoxins but also the infiltrations of neutrophils and monocytes, which further cause neuron damage, significantly amplifying the effects of ischemia and making the injury irreversible (Jiao et al., 2011; Obermeier et al., 2013; Sladojevic et al., 2019).

Oxidative stress is the major cause of BBB damage in ischemia that refers to a state in which the generation of reactive oxygen species (ROS) exceeds far behind the body’s dispose ability, leading to serious impairments (Kuźma et al., 2018; Chen et al., 2020). Redressing this imbalance between ROS and ROS scavenging in the brain vascular endothelial cells is necessary and urgent for stroke treatment. In comparison to directly eliminating ROS, inspiring the innate antioxidation system is a better choice as it has a higher efficiency, longer effecting duration, and more safety (Kuźma et al., 2018; Chen et al., 2020).

The nuclear factor erythroid 2–related factor 2 (Nrf2) belongs to the cap “n” collar (Cnc)-bZIP (basic leucine zipper) family and is a transcription factor regulating the expressions of a series of antioxidant, anti-inflammatory, and detoxifying proteins (Loboda et al., 2016). The Nrf2 pathway is the most important antioxidation machinery of the body and is also closely related to cerebral vascular endothelium preservation and the outcome of brain ischemia.

A large number of studies showed that the activation of the Nrf2 pathway can lead to strong antioxidative and antiapoptotic effects and BBB protection in the brain infarction (Zhao et al., 2007; Nguyen et al., 2009; Zhang et al., 2017; Hu et al., 2018; Li et al., 2018; Wang et al., 2018; Liu et al., 2019). In contrast, mass reports exhibited that the deletion or downregulation of Nrf2 exacerbated brain injuries in ischemia, in which the acceleratedly destroyed TJs in cerebral blood vessels, increased BBB breakdown, and brain edema played a pivotal role (Zhao et al., 2007; Li et al., 2014). These results indicate that the Nrf2 pathway is a pivotal target for vascular endothelial cell protection and stroke therapy.

SaiLuoTong capsule (SLT) is an outstanding representative of new type Chinese herbal preparation, which is composed of refined herbal extracts, instead of crude drugs, thus having a definite and controllable composition, in which the high efficient ingredients are enriched, and the lower and even inefficient ones are removed. This characteristic renders SLT to overcome the shortcomings of traditional Chinese herbal preparations, significantly enhances the controllability in production and safety, and increases the therapeutic effects.

SLT is composed of extracts of three Chinese herbal medicines, that is, the roots of *Panax ginseng* (ginseng), the leaves of *Ginkgo biloba* (ginkgo), and the flowers of *Crocus sativus* (saffron), with the proportion of 5:5:1 that is derived from pharmacodynamic optimization experiments in animals (Jia et al., 2018; Steiner et al., 2018). The main active components of SLT are ten compounds, including three ginsenosides, three flavones, three ginkgolides, and one crocin (Jia et al., 2018).

Numerous studies have testified the remarkable protective effect of SLT against brain ischemia. And the therapeutic mechanism was revealed as anti-inflammation, antioxidative stress, and antiapoptotic and platelet aggregation, as well as improving blood flow and brain tissue acetylcholine (ACh) content (Xu et al., 2008; Seto et al., 2017; Steiner et al., 2018; Zhang et al., 2019; Fan et al., 2020). More encouragingly, in a strictly designed clinical trial with multiple centers, large sample, and double-blinded placebo control, SLT showed a significant ameliorative effect in patients with mild to moderate vascular dementia; meanwhile, no significant toxicities were exhibited (Jia et al., 2018; Steiner et al., 2018). At present, an internationally cooperated phase III clinical trial about the effects of SLT on brain ischemia–related cognition impairments is well ongoing in both China and Australia. Thus, SLT has a good chance to be accepted as an official drug for the remedy of stroke and its sequelae by the international mainstream, which will possibly be the first worldwide applied Chinese herbal drug in the major diseases, not only being a milestone of Chinese medicine but also bringing a light to the unsatisfying situation of stroke remedy.

Therefore, further exploring SLT’s therapeutic mechanisms has a particular significance and should be a long-lasting issue. The relationship between SLT’s anti-stroke effect and Nrf2 pathway–mediated cerebral vascular endothelial cell protection has not been clarified. In the present study, we investigated this issue with experiments on both rats and cultured cells. This study may be significant for clarifying the therapeutic mechanism of SLT on stroke and for searching effective drugs for stroke remedy.

## Materials and Methods

### Animal Preparation

Male Sprague–Dawley rats (200–230 g) were purchased from SPF Biotechnology Co., Ltd. (Beijing, China, No. 1103241911033018). Rats were maintained in an air-conditioned room (temperature: 21 ± 2°C) under a 12 h day–night cycle with free access to food and water, and they were acclimated for 3 days prior to the experiment. Animal handling procedures were performed in accordance with the guide of the Ethics Committee of Xi Yuan Hospital of China Academy of Chinese Medical Sciences (Protocol No. 2019XLC015-2). And all animal housing, care, feeding, and experimental procedures were in compliance with the National Guidelines for Animal Protection.

### Establishment of Cerebral Infarction Model in Rats With Permanent Middle Cerebral Artery Occlusion

Rats were anesthetized by an intraperitoneal injection with 1% pentobarbital sodium (80 mg/kg). Under anesthesia, the right common carotid, the right external carotid, and the right internal carotid were carefully separated and exposed. The right external carotid and the right common carotid were ligated with a suture silk. Thereafter, a 3–0 monofilament nylon suture with a rounded tip of 0.32 mm diameter (Item#2432A1, Beijing Sunbio Biotech Co Ltd.) was introduced into the bifurcation of the right common carotid and then was intracranially inserted for approximately 18 mm to block the blood flow of the right middle cerebral artery. During this procedure, the body temperature was maintained at 37°C using a warm pad. For the sham-operated group, only skin incisions were performed under anesthesia.

### Drug Treatments in Rats

SLT was provided by the ShenWei Pharmaceutical Corporation (Heibei, China). SLT was soluted in saline and was injected into the duodenum immediately after the right middle cerebral artery was blocked. The doses of SLT were set as 16.5 mg/kg (SLT-L) and 33 mg/kg (SLT-H). Rats in the sham group and model group were injected with saline in the same way and at the same time point.

### Measurement of Neurological Deficits

The neurological function deficit scores of rats were blindly evaluated 24 h after MCAO. A five-point scale was used as follows: 0, no neurological deficits; 1, failure to fully extend the left forelimb; 2, decreased resistance to a lateral push toward the right side and failure to fully extend the left forepaw; 3, circling to the left side; and 4, inability to walk spontaneously and lack of response to stimulation (Bederson et al., 1986).

### Assessment of Infarct Volumes

After neurological function tests, the rats were killed by decapitation, and their brains were taken out and were sectioned into slices of 2 mm thickness; the slices were incubated in a 2% 2,3,5-triphenyltetrazolium chloride (TTC) solution at 37°C for 15 min. TTC stained the non-infarcted region with a deep red pigment, while the infarcted brain areas were stained with white (Bederson et al., 1986). Stained sections were photographed, and the images were analyzed to calculate the infarct volumes by using an Image Pro-Plus 6.0 analysis system (Media Cybernetics, Rockville, MD, United States).

### Brain Tissue Fixation, Embedding, and Sectioning, and HE Staining

24 h after MCAO, the rats were killed by decapitation, and the brains were then rapidly taken out and were placed in 4% paraformaldehyde for 7 days. The brains were then embedded in paraffin and were sectioned into slices of 7 µm thickness. The sections were stained with HE and were observed using an Olympus BX51 microscope.

### Evaluation of Water Content of the Brain

24 h after MCAO, rats were sacrificed by decapitation, and their brains were taken out. The wet brains were weighed and were dried at 60°C for 3 days, and then the weights of the dry brains were measured. The brain water content = (1-dry weight/wet weight)×100%.

### Immunohistochemistry Examination

The brain sections were deparaffinized, rehydrated, and blocked. Next, the sections were incubated overnight at 4°C with anti–claudin-1 antibody (1:500, ab15098, abcam), anti-occludin antibody (1:500, 27260-1-AP, ProteinTech), and anti–heme oxygenase-1 (HO-1) antibody (1:500, 66743-1-Ig, ProteinTech). The sections were rinsed with PBS three times, then were incubated with horseradish peroxidase (HRP)-conjugated goat anti-rabbit IgG at 37 °C for 20 min, and were colorized with DAB. Last, hematoxylin restaining was performed. The sections were observed using an Olympus BX51 microscope.

### Western Blot Assay

The nuclear and cytosol protein extractions and total protein extractions from the samples (in *in vivo* experiments, were cerebral cortex; in *in vitro* experiment, were hCMEC/D3 cells) were performed using the nuclear-cytosol extraction kit (Applygen Technologies Inc., Beijing) and the total protein extraction kit (Solarbio Science and Technology Co. Ltd., Beijing), respectively. Equal amounts of protein (50 µg) were loaded into 10% or 12.5% SDS-PAGE gels, and then were subjected to electrophoresis; last, they were transferred to nitrocellulose membranes (Millipore, Billerica, MA, United States). The membranes were blocked with 5% bovine serum albumin (BSA) for 1 h at room temperature, and then were incubated overnight at 4°C with anti–claudin-1 antibody (1:1000), anti-occludin antibody (1:1000), anti-Nrf2 antibody (1:1000), anti–HO-1 antibody (1:1000), anti-GAPDH antibody (1:1000,60004-1-Ig, ProteinTech), anti–β-actin antibody (1:1000, 60008-1-Ig, ProteinTech), or anti–histone H3 antibody (17168-1-AP, ProteinTech). The membranes were incubated with HRP-conjugated secondary antibodies for 1.5 h at room temperature. The protein bands were enlightened with an enhanced chemiluminescence kit, and their brightness was quantified by using Image LabTM software.

### Determinations of the Activities of Superoxide Dismutase and the Contents of Glutathione

All samples (brains and cells) were made into homogenates by using an ultrasonic cell disrupter at 0°C. SOD activities and GSH contents in the homogenates were analyzed with the merchant kits (Institute of Biological Engineering of Nanjing Jiancheng, Nanjing, China).

### Cell Culture

Human brain microvascular endothelial cell lines (hCMEC/D3, iCellBioscience, Inc. Shanghai, China) were cultured at 37 °C with 5% CO_2_ in the endothelial cell medium (ECM, PriMed-iCell-0016, China) supplemented with 5% fetal bovine serum, 1% ECGS, 100 U/mL penicillin, and 100 μg/ml streptomycin, and were passaged with 0.25% trypsin.

### Oxygen-Glucose Deprivation and Reoxygenation Model and SLT Treatments

For oxygen-glucose deprivation (OGD), hCMEC/D3 cells were incubated with a glucose-free Dulbecco’s modified Eagle’s medium (DMEM, Gibco, United States) and were placed in a customer-made chamber, which was then filled with 95% N_2_/5% CO_2_ and kept at 37°C for 4 h. After OGD, for reoxygenation, the incubation media were replaced with the normal ECM, and the cells were cultured under normal atmosphere with 5% CO_2_. The mock group was incubated in normal DMEM under normal atmosphere with 5% CO_2_ at 37°C for 4 h and was then incubated with ECM. SLT was soluted with DMSO, and the solutions were added into the incubation medium with the volume ratio of 1:1000 at the beginning of OGD and reoxygenation. The mock group and the OGD/R model group were treated with DMSO in the same way at the same time points.

### Cell Viability Measurement

The cells were incubated with CCK8 (Dojindo Corporation, Japan)-ECM solution (1:10) at 37°C for 2 h. The absorbance at 450 nm was measured using a microplate reader. Then the cell viabilities were obtained through normalization to the average absorbance of the mock (normal control) group.

### Immunofluorescence Assay

Paraformaldehyde-fixed hCMEC/D3 cells were incubated with anti-Nrf2 antibody or anti–HO-1 antibody (both 1:200), followed by the incubation with secondary antibodies, that is, fluorescein-conjugated AffiniPure goat anti-rabbit IgG (1:100, Yuabio, China), and were stained with DAPI finally. Cells were observed and imaged using an Olympus IX81 live cell station.

### RNA Interference of Nrf2

Transfections in HCMEC/D3 cells were conducted with Lipofectamine 3000 reagent (Thermo Fisher Scientific, United States). High purity control siRNAs (negative control siRNA and GAPDH siRNA) and Nrf2 siRNAs were obtained from JTSBIO (Wuhan, China). The Nrf2 siRNA sequences were as follows: Nrf2 siRNA-1, forward, CCC​UGA​AAG​CAC​AGC​AGA​ATT, and reverse, UUCUGCUGUGCUUUCAGGGTT; Nrf2 siRNA-2, forward, CCA​GAA​CAC​UCA​GUG​GAA​UTT, and reverse, AUU​CCA​CUG​AGU​GUU​CUG​GTT; and Nrf2 siRNA-3, forward, GCCUGUAAGUCCUGGUCAUTT, and reverse, AUG​ACC​AGG​ACU​UAC​AGG​CTT. Negative control (NC) siRNA sequences were as follows: forward, UUC​UCC​GAA​CGU​GUC​ACG​UTT, and reverse, ACG​UGA​CAC​GUU​CGG​AGA​ATT. GAPDH sequences were as follows: forward, UGA​CCU​CAA​CUA​CAU​GGU​UTT, and reverse, AAC​CAU​GUA​GUU​GAG​GUCAT​T.

### Paracellular Permeability Measurement

Cell monolayer integrity was assessed by diffusion of fluorescein isothiocyanate (FITC)-dextran (4kDa, Lot: 64,878, MCE, United States) as previously described (Hu et al., 2018). After OGD/R, 400 μL FITC-dextran (0.5 mg/ml) solutions were added to the upper chamber of the 12-well transwell culture plate inserts (A190059, Millicell, Germany) on the bottom of which the hCMEC/D3 cell confluents grew. Inserts were placed in the 12-well culture plates containing 1000 μL of DMEM/F12 media (without serum and phenol red) per well. Then the cells were incubated at 37°C for 60 min in the dark. Inserts were removed, and the solutions in the wells were collected and transferred into a black 96-well plate. The fluorescence intensities were measured by using the excitation and emission wavelengths at 490 and 520 nm, respectively, and were converted to the concentrations of FITC-dextran with the calibration curve.

### Statistical Analysis

The data are expressed as mean ± SD and were statistically analyzed by using the Statistical Product and Service Solutions (SPSS) 16.0 software; data comparisons between two groups were conducted with the t-test, and those among multiple groups were conducted with one-way or two-way analysis of variance (ANOVA) followed by the LSD test. The statistical significance level was set to 0.05.

## Results

### SLT Attenuated Cerebral Infarctions and Improved the Neurological Functions in MCAO Rats

**FIGURE 1:**
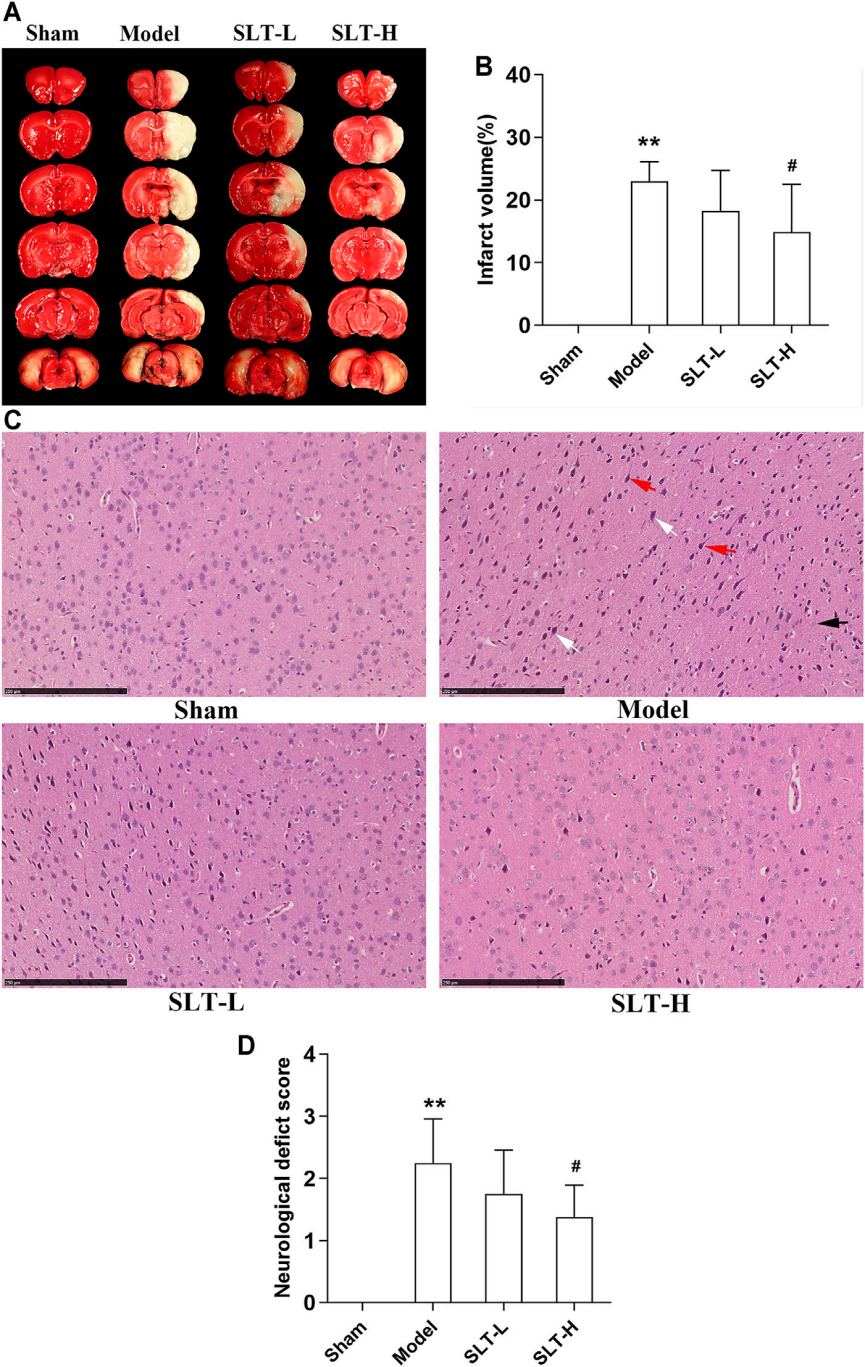
SLT attenuated cerebral infarction and neurological deficit in rats undergoing MCAO. 24 h after MCAO, the rats were neurologically tested, then were killed, and their brains were harvested for TTC and HE stainings. **(A)** Representative images of TTC staining. The white regions are the infarct tissues, while the red regions are the viable tissues. **(B)** Infarct volumes of the brains (*n* = 8). **(C)** Representative images of HE staining of the brain cortex in all the groups. The ischemic brain region in the model group presented severe neuronal loss and neuron degeneration, including cytoplasm acidophilic degeneration (indicated with white arrows), nuclear pyknosis (indicated with red arrows), and hydropic degeneration (indicated with black arrow). The neuronal damages were alleviated in SLT-L and SLT-H groups (scale bar in all panels = 250 μm). **(D)** Neurological deficit scores of all the groups (*n* = 8). Data are expressed as mean ± SD. *p < 0.05, **p < 0.01, in comparison to the sham group; #*p* < 0.05, in comparison to the model group.

No infarction was detected in the sham group, while infarctions of large volumes were developed in the model group. SLT treatments alleviated the brain infarctions, and the infarct volumes in the SLT-H group were significantly lower than those in the model group (*p* < 0.05, Figures 1A,B). Accordingly, in the sham group, no neuron damage, neuron loss, or other notable morphological abnormalities were shown in the brain microscopy; however, necrosis of large areas was observed in the model group, in which severe neuron loss, neuron cell degeneration including cytoplasm acidophilic degeneration, and nuclear pyknosis were shown; tissue edema and neuron hydropic degeneration were shown in the regions peripheral to necrosis, and these lesions were notably attenuated in SLT-treated groups, especially in the SLT-H group (Figure 1C).

Neurological deficit scores were significantly elevated in the model group in comparison to the sham group (*p* < 0.01), indicating a severe injury in neurological function was induced by brain ischemia. However, treatment with SLT alleviated the elevation in neurological deficit scores, especially with the high dose, of which the effect was significant (*p* < 0.05, compared with the model group; Figure 1D).

### SLT Decreased Water Content of the Brain and Inhibited the Drop in Expression Levels of Claudin-1 and Occludin in MCAO Rats

**FIGURE 2:**
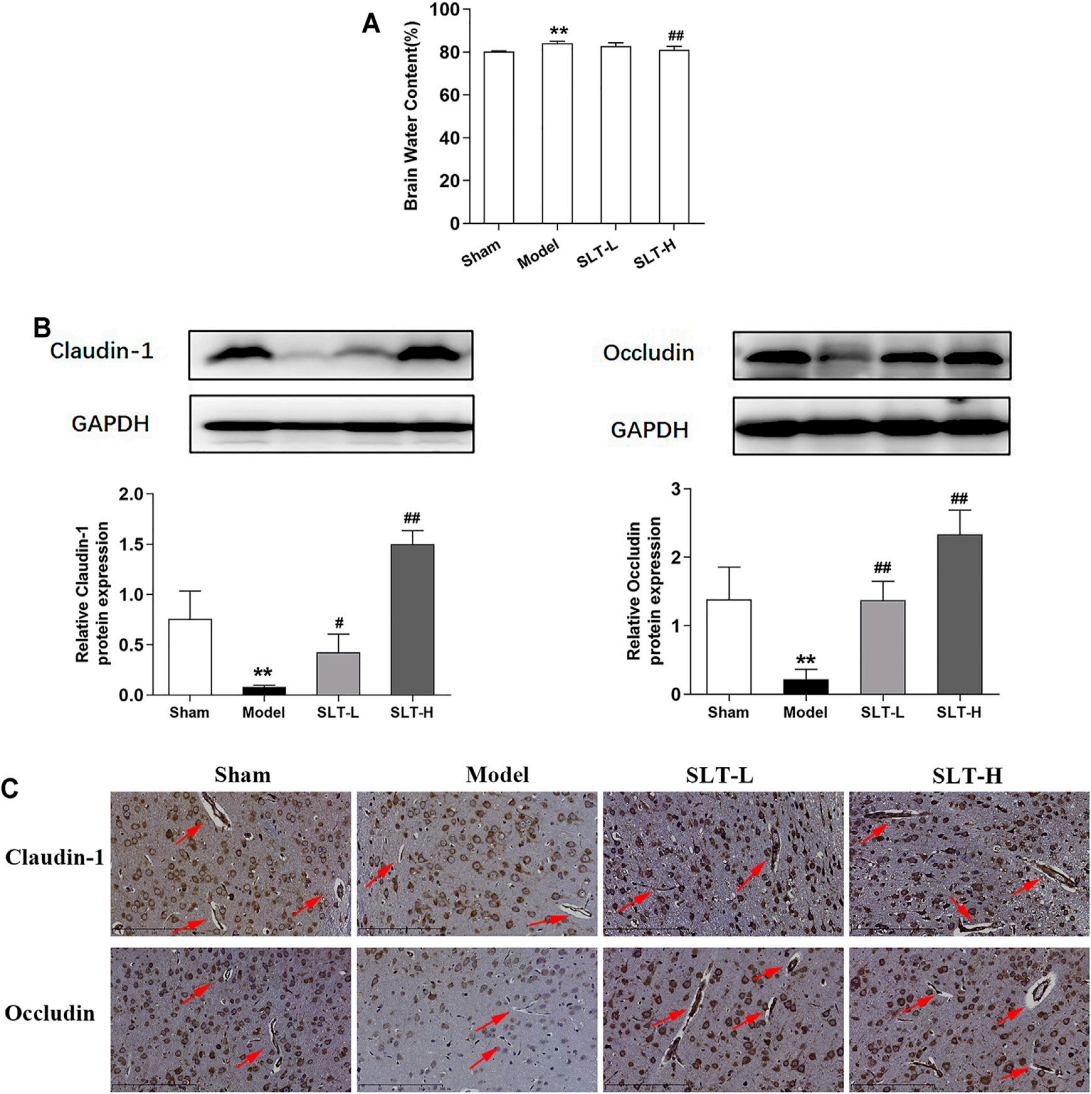
SLT decreased brain edema and prevented the loss of tight junction proteins in the cerebral vascular endothelium in MCAO rats. **(A)** Brain water contents of the ischemic hemispheres measured by the wet and dry weight method (*n* = 7). **(B)** Western blot assays for claudin-1 and occludin expressions in the ischemic hemispheres (*n* = 3). **(C)** Representative images of immunohistochemistry examination for claudin-1 and occludin in the ischemia areas. The arrows point to the blood vessels (scale bar = 200 μm). Data are represented as mean ± SD. *p < 0.05, **p < 0.01, compared with the sham group; #*p* < 0.05, ##*p* < 0.01, compared with the model group.

The water contents of the infarcted hemispheres in the model group were significantly increased in comparison to the ipsilateral hemispheres in the sham group (*p* < 0.01); however, water contents of the infarcted hemispheres in SLT groups were decreased, especially in the SLT-H group, which were significantly lower than those in the model group (*p* < 0.01, Figure 2A). These results hint that SLT may have the effect of protecting cerebral microvascular, thus alleviating its leakage.

Additionally, the examinations on claudin-1 and occludin, two key TJ-related proteins that play pivotal roles in maintaining BBB integrity, showed similar results. The results of the Western blot assay showed that the expression levels of claudin-1 and occludin in the infarcted hemispheres significantly decreased in the model group, compared with the ipsilateral hemispheres in the sham group (*p* < 0.01); however, the expression levels of claudin-1 and occludin were significantly increased in SLT-administrated groups in comparison to the model group (*p* < 0.05 or *p* < 0.01, Figure 2B). Immunohistochemistry examination results showed that the expression levels of claudin-1 and occludin in cerebral microvascular endothelial cells in the infarcted area were attenuated in the model group, in comparison to the same positions in the sham group; however, these downregulations in claudin-1 and occludin expressions were prevented by SLT treatments (Figure 2C).

Collectively, these results hint that the cerebral microvascular may be a key target of SLT, and the protection on them may constitute the foundation for SLT’s therapeutic effects on brain ischemia.

### SLT Activated the Nrf2 Pathway in the Brain of MCAO Rats

**FIGURE 3:**
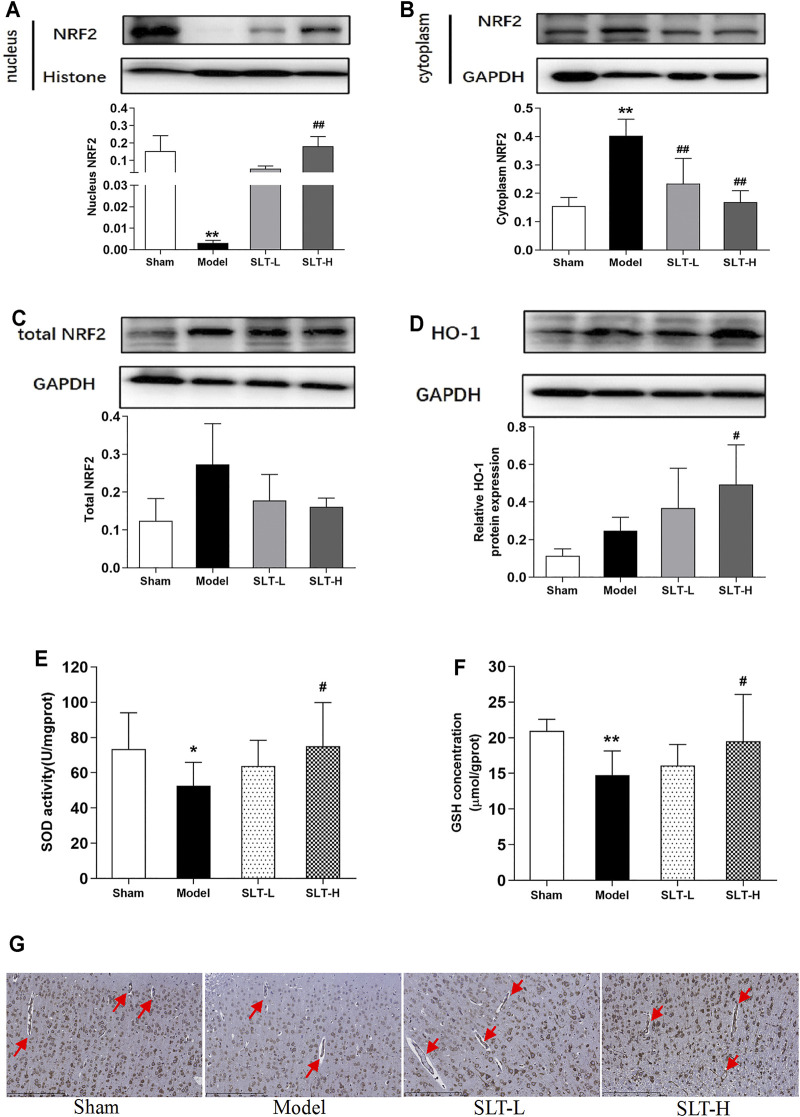
SLT activated the Nrf2 pathway in the brain after ischemia. **(A–C)** Western blot assays for the protein levels in the ischemic hemispheres of nucleus Nrf2, cytoplasm Nrf2, and the total Nrf2 (*n* = 3). **(D)** HO-1 expressions in the ischemic hemispheres. **(E)** and **(F)** SOD activities and GSH contents in the ischemic hemispheres (*n* = 8). **(G)** Immunochemistry examination of HO-1 expressions in the ischemic regions. Red arrows indicate the blood vessels (scale bar = 250 μm). Data are expressed as mean ± SD. *p < 0.05, **p < 0.01, vs. the sham group; #*p* < 0.05, ##*p* < 0.01, vs. the model group.

The nuclear contents of Nrf2 in the infarcted hemispheres in the model group were significantly lower than those in the ipsilateral hemispheres in the sham group (*p* < 0.01); correspondingly, Nrf2 contents in the cytoplasm of the model group were significantly higher (*p* < 0.01). This result indicates that after MCAO, the Nrf2 pathway was drastically compromised in rat brains. However, SLT treatments rescued this fall down; in SLT-H groups, Nrf2 levels in the nucleus were significantly increased (*p* < 0.01, compared with the model group), and the contents of Nrf2 in the cytoplasm of SLT-L and SLT-H groups were significantly lower than those in the model group (both *p* < 0.01, Figures 3A,B). Besides, the total protein levels of Nrf2 between all the groups showed no significant difference, suggesting the changes in the Nrf2 pathway were mainly derived from posttranslational modifications, which are also the major regulation fashion of the Nrf2 pathway (Figure 3C).

The downstream of Nrf2 exhibited similar changes. Treatments with SLT increased the protein expressions of HO-1 in the ischemia hemisphere, especially with SLT-H, which showed a significance in statistics (compared with the model group, *p* < 0.05, Figure 3D). Immunohistochemistry examination revealed that the upregulation of HO-1 by SLT treatments also happened in cerebral vascular endothelial cells (Figure 3G). The activities of SOD and the contents of GSH in the infarcted hemispheres in the model group were also significantly decreased in comparison to the ipsilateral hemispheres in the sham group (*p* < 0.05 in SOD activities and *p* < 0.01 in GSH contents), and these decreases were inhibited in SLT treatment groups as well, with better effects in the SLT-H group, which has statistical significances in comparison to the model group (*p* < 0.05 in SOD activities and *p* < 0.05 in GSH contents, Figures 3E,F).

Taken together, these results showed SLT treatments activated the Nrf2 pathway in the brain and imply that it may play a key role in SLT’s therapeutic effects on brain ischemia including the protection of cerebral microvascular endothelium.

### SLT Protected hCMEC/D3 Cells and Activated the Nrf2 Pathway in hCMEC/D3 Cells in OGD/R Injury

The above results of *in vivo* experiments suggest that SLT may exert its anti-brain ischemia effect through cerebral microvascular protection, which may be related to the activation of the Nrf2 pathway. To confirm this assumption, we selected a human brain microvascular endothelial cell line, hCMEC/D3, and observed the direct effect of SLT on it.

**FIGURE 4:**
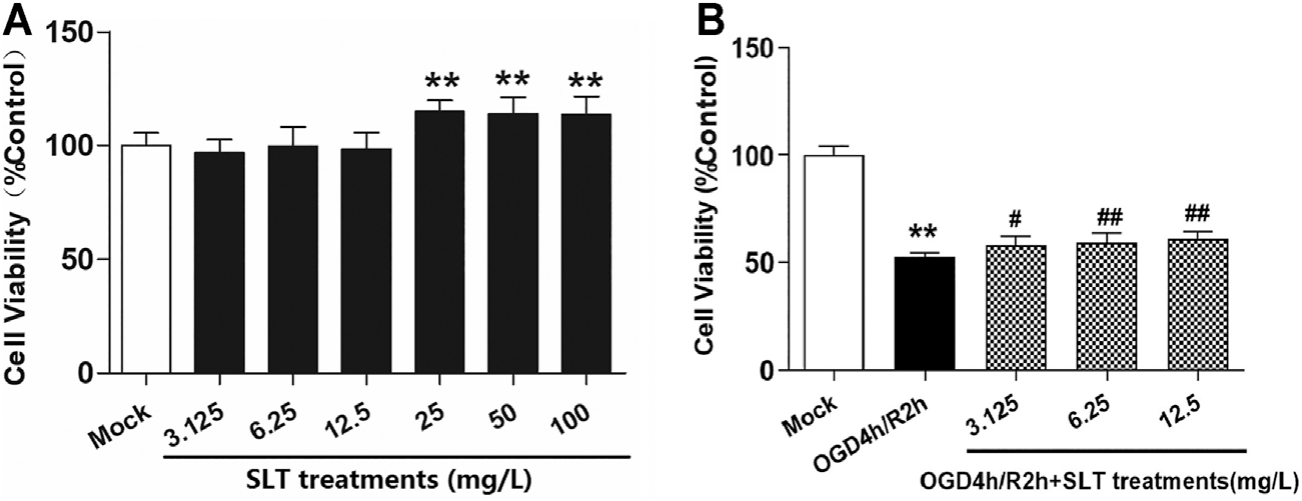
SLT alleviated OGD/R injury in hCMEC/D3 cells. **(A)** Toxicity test of SLT on hCMEC/D3 cells (*n* = 6). The cells were cultured with different concentrations of SLT (3.125, 6.25, 12.5, 25, 50, and 100 mg/L) for 24 h, and the cell viabilities were examined by the CCK-8 method. **(B)** The therapeutic effects of SLT on OGD/R injury in hCMEC/D3 cells (*n* = 6). Data are expressed as mean ± SD. *p < 0.05, **p < 0.01, vs. the mock group; #*p* < 0.05, ##*p* < 0.01, vs. the OGD/R group.

First, we observed the safety range of SLT on normal hCMEC/D3 cells with the CCK8 test. In the concentration range of 3.125–100 mg/L, SLT did not significantly decrease the cell viabilities; on the contrary, at concentrations of 25, 50, and 100 mg/L, SLT significantly increased the cell viabilities (*p* < 0.01, compared with the mock group; Figure 4A). This result indicates that up to 100 mg/L, SLT has no toxicities on hCMEC/D3 cells because there was no significant decline shown in cell viabilities.

Second, we duplicated the OGD/R model in hCMEC/D3 cells, which simulates ischemia injury *in vivo*, and treated the cells with SLT. The results showed that OGD/R injury significantly decreased the cell viabilities (*p* < 0.01, compared with the mock group); however, SLT treatments at concentrations of 3.125, 6.25, and 12.5 mg/L significantly increased the cell viabilities (all *p* < 0.01, compared with the model group, Figure 4B), confirming that SLT has a direct protective effect on microvascular endothelial cells against ischemia assault.

**FIGURE 5:**
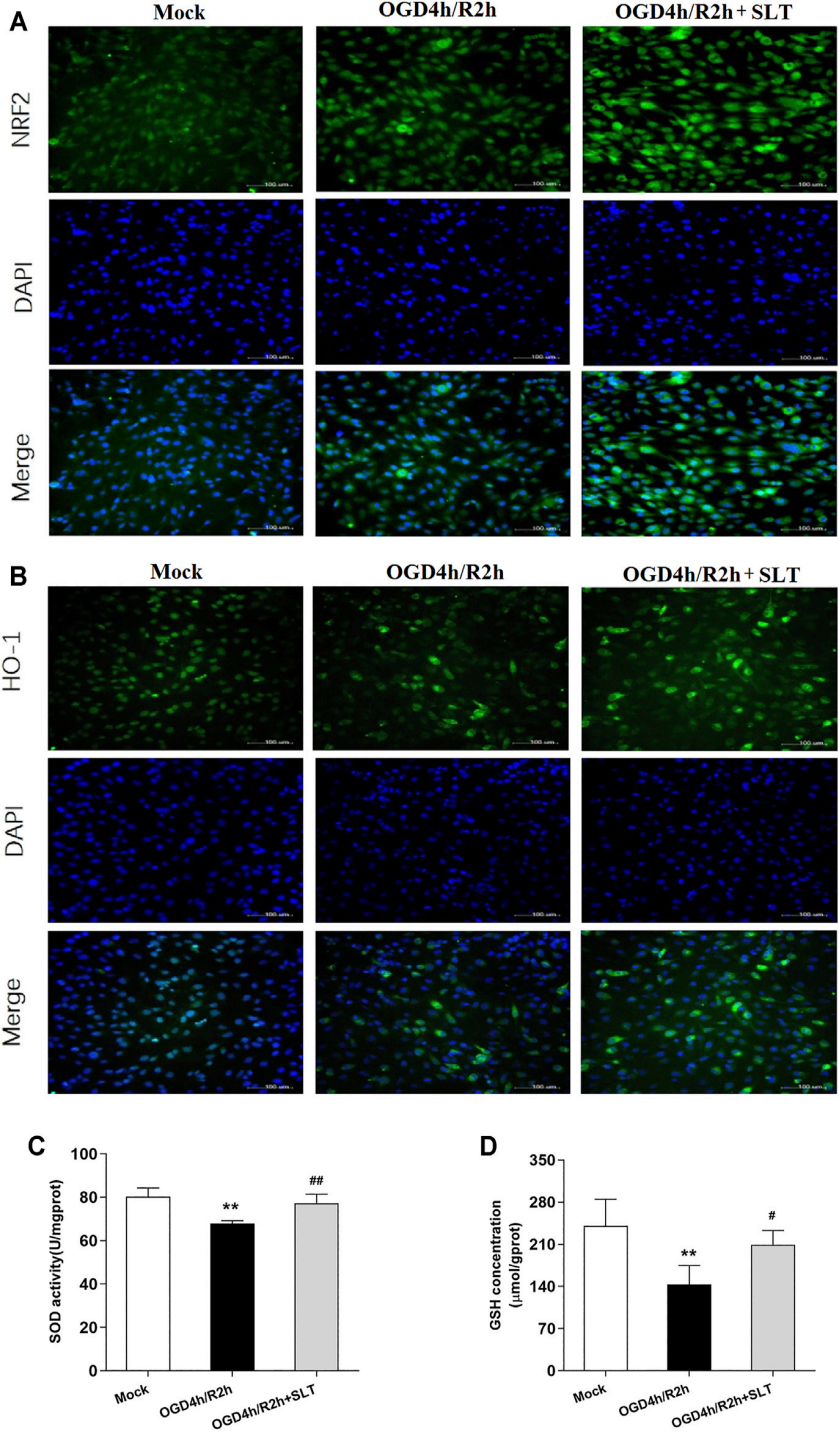
SLT activated Nrf2 pathway in OGD/R-injured hCMEC/D3 cells. **(A,B)** Images of cytoimmunofluorescent examination of Nrf2 and HO-1 expressions in cells undergoing OGD/R injury (scale bar = 100 μm). **(C,D)** SOD activities and GSH contents in hCMEC/D3 cells undergoing OGD/R injury (*n* = 5 each). Data are expressed as mean ± SD. *p < 0.05, **p < 0.01, vs. the mock group; #*p* < 0.05, ##*p* < 0.01 vs. the OGD/R group.

Third, we investigated the activities of the Nrf2 pathway in SLT’s intervening. We selected the medium effective concentration, that is, 6.25 mg/L, of SLT to treat hCMEC/D3 cells. The results of immunocytofluorescent examinations showed that cells in the mock group exhibited weak expressions of Nrf2 and HO-1; in contrast, cells in the model group displayed slightly enhanced expressions. However, the expressions of these two proteins in cells of SLT-treated groups were strongly enhanced in comparison to the model group; furthermore, the enhancedly expressed Nrf2 was mainly nuclear distributed (Figures 5A,B). SOD activities and GSH levels in hCMEC/D3 cells showed an accordant situation. They were significantly decreased in the OGD/R group (*p* < 0.01, compared with the mock group); however, compared with the model group, the SOD activities and GSH levels in the SLT-treated group were significantly increased (*p* < 0.01, Figures 5C,D). This result directly testified that SLT treatment activates the Nrf2 pathway in microvascular endothelial cells and further links the Nrf2 pathway to SLT’s vascular endothelium protection.

### Nrf2 siRNA Interference Inhibited SLT’s Protective Effect Against OGD/R Injury in hCMEC/D3 Cells

To finally confirm the role of the Nrf2 pathway in SLT’s therapeutic effect, we performed a counterevidence experiment.

**FIGURE 6:**
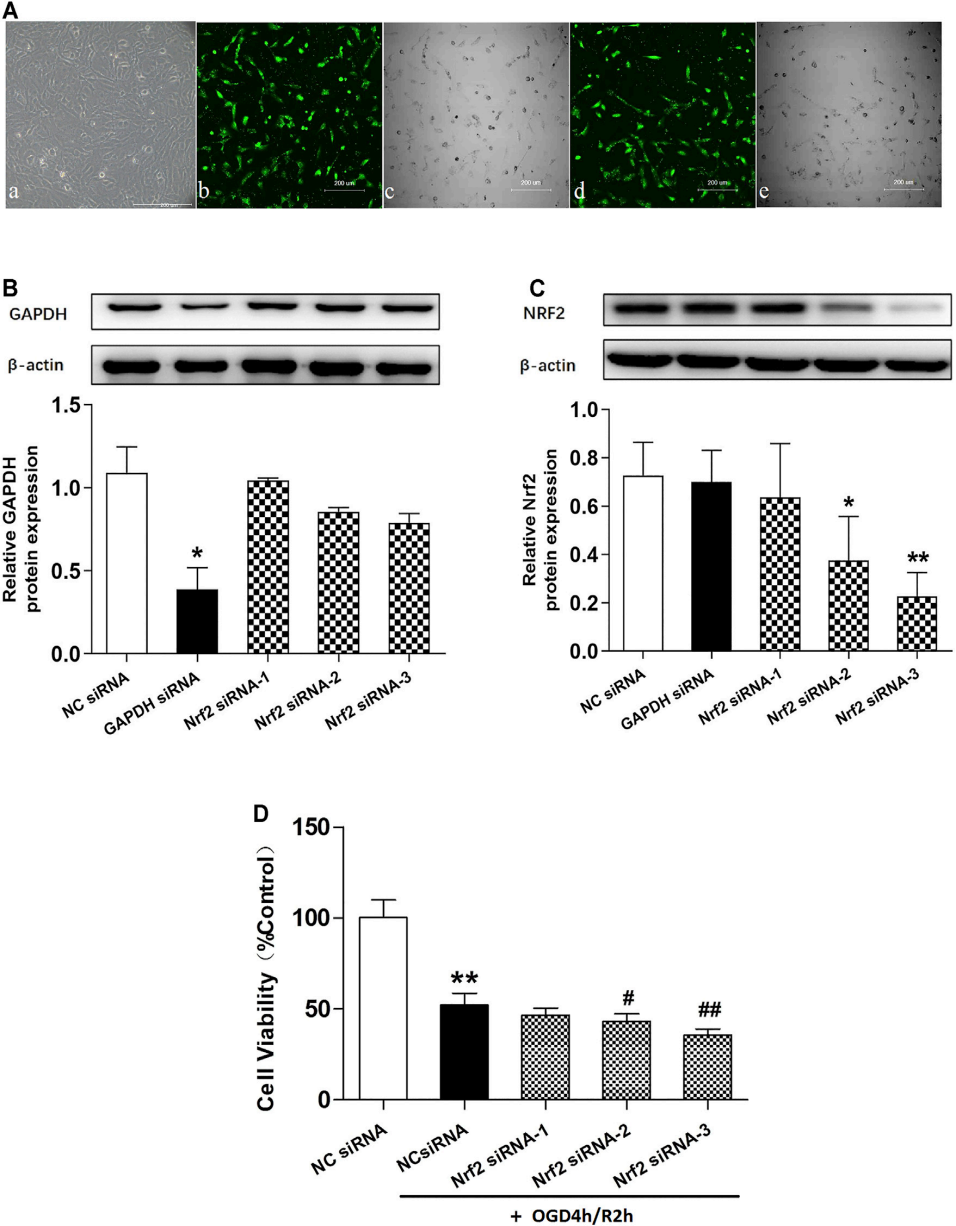
Effects of different Nrf2 siRNA interferences on Nrf2 expressions and hCMEC/D3 cell viabilities in OGD/R. **(A)** (a) hCMEC/D3 cells under normal culture condition. (b,c,d,e) Different concentrations of the negative control siRNA labeled with green fluorescence were applied to hCMEC/D3 cells to help optimize the transfection conditions. (b,c) Fluorescence and bright field images of hCMEC/D3 cells treated with 50 nmol/L plasmid. (d,e) Fluorescence and bright field images of hCMEC/D3 cells treated with 100 nmol/L plasmid. Plasmids of the two concentrations showed similar transfection efficiency, while the lower one showed less cell loss; thus, 50 nmol/L was selected as the optimal concentration for transfection and was used in the subsequent experiments. **(B,C)** Western blot assays for expressions of GAPDH and Nrf2 in hCMEC/D3 cells. The cells were transfected with GAPDH siRNA plasmid or Nrf2 siRNA plasmid (Nrf2 siRNA-1, Nrf2 siRNA-2, and Nrf2 siRNA-3); the results showed the expressions of target genes were specifically knocked down by aiming siRNAs, testifying specificities and efficiencies of the siRNAs. Besides, Nrf2 siRNA-3 showed the strongest ability to interfere with the expression of Nrf2. **(D)** Effects of different Nrf2 siRNAs on OGD/R injury of hCMEC/D3 cells. Data are expressed as mean ± SD (*n* = 3). *p < 0.05, **p < 0.01 vs. negative control siRNA (NC siRNA); #*p* < 0.05, ##*p* < 0.01 vs. NC siRNA + OGD/R.

First, we constituted siRNAs to knock down Nrf2. The optimal transfection concentration of plasmid that leads to minimum cytotoxicity and can efficiently downregulate target gene expressions was determined as 50 nmol/L (Figure 6A). Then, three Nrf2 siRNA (siRNA-1, siRNA-2, and siRNA-3) and negative control siRNA (NC siRNA) sequences were designed, synthesized, and transfected into hCMEC/D3 cells. The results showed that Nrf2 siRNA-3 had the strongest ability to inhibit Nrf2 protein expression, and interestingly, this siRNA also significantly decreased the cell viability in OGD/R injury, which is agreed with the well-recognized safeguard role of the Nrf2 pathway in ischemia injury (*p* < 0.01, compared with NC siRNA + OGD/R group, Figures 6B–D). Therefore, Nrf2 siRNA-3 was selected to study the role of Nrf2 in SLT’s therapeutic effect on OGD/R injury of hCMEC/D3 cells.

**FIGURE 7:**
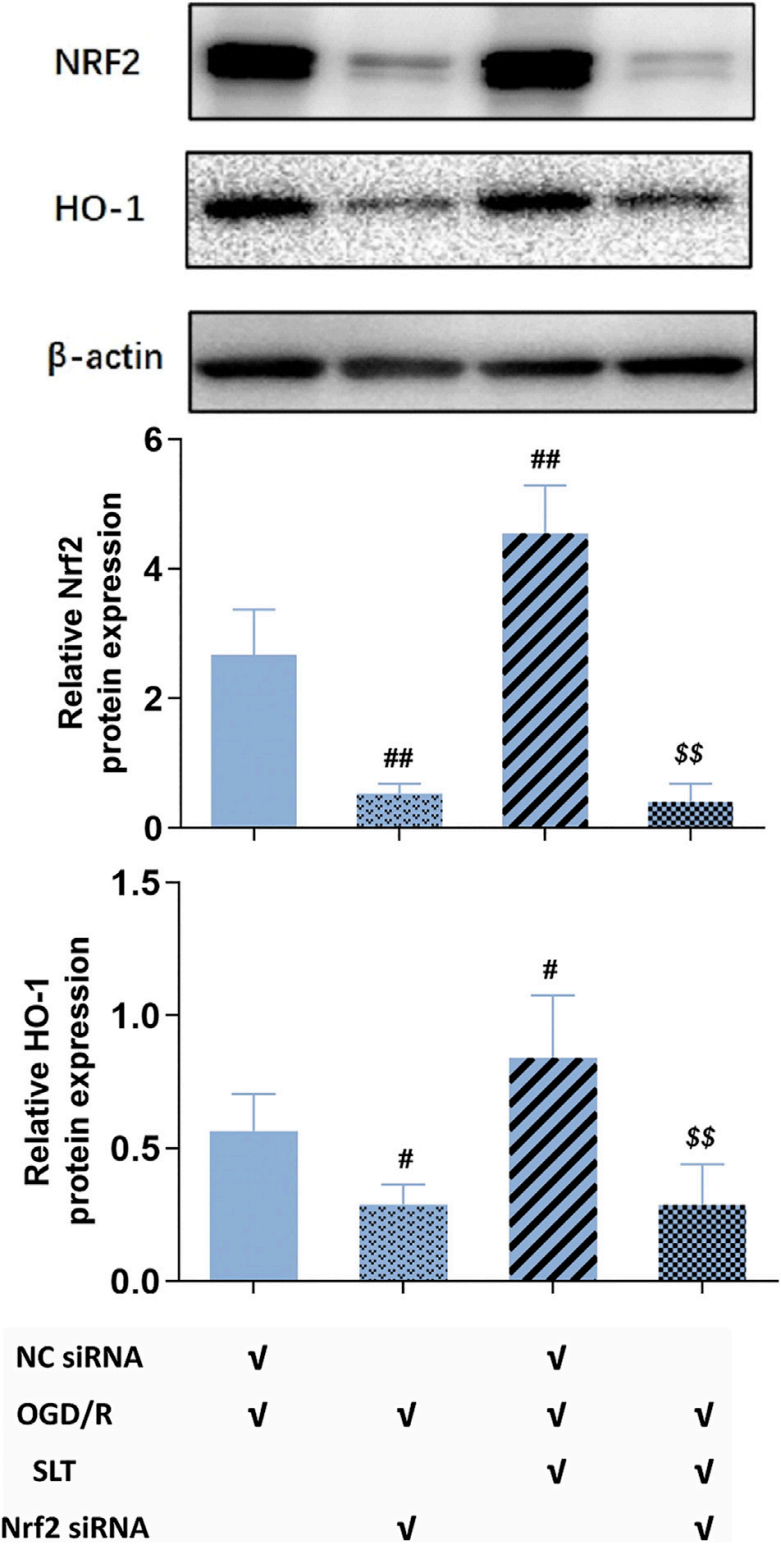
Nrf2 siRNA abrogated activation of SLT on the Nrf2 pathway in cerebral microvascular endothelial cells in OGD/R injury. Data are expressed as mean ± SD (*n* = 3). #*p* < 0.05, ##*p* < 0.01 vs. NC siRNA + OGD/R group; $ *p* < 0.05, $$ *p* < 0.01, vs. NC siRNA + OGD/R + SLT group.

**FIGURE 8:**
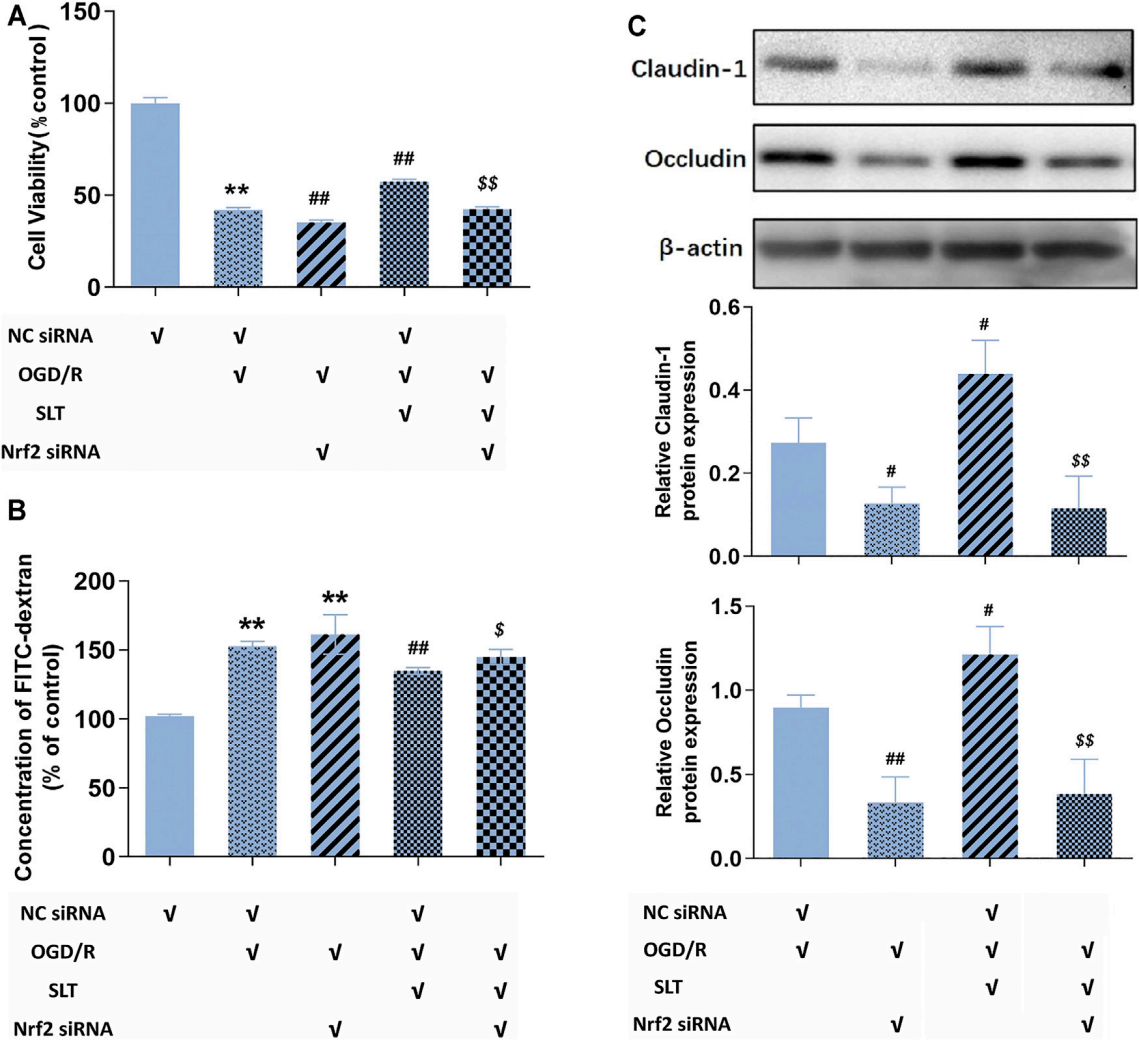
Blocking of the Nrf2 pathway ablated SLT’s protection against OGD/R injury in cerebral microvascular endothelial cells. **(A,B)** Effects Nrf2 siRNA interference on SLT’s ameliorations on cell viabilities and FITC-dextran permeabilities in OGD/R-injured hCMEC/D3 cells (*n* = 6). **(C)** Effects of Nrf2 siRNA interference with SLT’s ameliorations on claudin-1 and occludin expressions in OGD/R-injured hCMEC/D3 cells (*n* = 3). Data are expressed as mean ± SD. *p < 0.05, **p < 0.01, vs. negative control siRNA (NC siRNA) group; #*p* < 0.05, ##*p* < 0.01, vs. NC siRNA + OGD/R group; $ *p* < 0.05, $$ *p* < 0.01, vs. NC siRNA + OGD/R + SLT group.

Next, we duplicated OGD/R injury in hCMEC/D3 cells, treated it with SLT, and observed the blocking effect of Nrf2 siRNA on SLT’s therapy. As expected, the elevations of protein expressions of Nrf2 and HO-1 by SLT treatment (*p* < 0.05 or 0.01, NC siRNA + OGD/R + SLT group vs. NC siRNA + OGD/R group) were significantly prevented by Nrf2 siRNA interference, indicating the Nrf2 pathway was specifically blocked (all *p* < 0.01, OGD/R + SLT + Nrf2 siRNA group vs. NC siRNA + OGD/R + SLT group, Figure 7). The alleviating effects of SLT on OGD/R injury in hCMEC/D3 cells include significantly increasing the cell viabilities, reducing endothelium permeability, and elevating expressions of claudin-1 and occludin (*p* < 0.05 or 0.01, NC siRNA + OGD/R + SLT group vs. NC siRNA + OGD/R group); however, these protective effects were all significantly inhibited by Nrf2 siRNA interference (*p* < 0.05 or *p* < 0.01, OGD/R + SLT + Nrf2 siRNA group vs. NC siRNA + OGD/R + SLT group; and *p* > 0.05, OGD/R + Nrf2 siRNA group vs. OGD/R + SLT + Nrf2 siRNA group; Figures 8A–C). This result undoubtedly indicates the protective effect of SLT on OGD/R injury of hCMEC/D3 cells is Nrf2 pathway dependent.

## Discussion

The mass and devastating neuron damages are the ultimate consequence of cerebral infarction; however, with the deepening of the research on the mechanism of brain ischemia, factors out of the neural cells deserve more and more attentions (Andjelkovic et al., 2019). Ischemia leads to a drastic drop in the supplies of oxygen and nutrients mainly including glucose, which injures neurons and also injures brain vascular endothelial cells at the same time (Brouns et al., 2009). The vascular endothelial cells exhibit severe cellular edema, that is, hydropic degeneration, even death, which results in disruption in the endothelium TJs, and attenuates its barrier function, leading to serious exudations from the blood vessels, which finally cause grievous edema and inflammation in the brain tissue, generating additive injuries to the neurons (Andjelkovic et al., 2019). These injuries secondary to ischemia are far more detrimental, both more violent and more extensive, which could expand to semi-ischemic and even non-ischemic regions, thus significantly amplifying the damages, even by fold (Brouns et al., 2009). Actually, most of the fatally devastating events elicited by cerebral infarction, such as cerebral edema, intracranial hypertension, cerebral hernia, coma, and even death, are largely related to vascular damages (Rahimi et al., 2017). Thus, the vascular factor plays a pivotal role in the pathogenesis of cerebral ischemia, even more important than ischemia itself.

The formation of endothelial TJs is dependent on a sort of specific proteins, of which claudin and occludin are two major members. These two proteins have a similar action mode; that is, the same kind of molecules in membranes of the two neighboring cells combine each other and form homological complexes, thus sealing off the intercellular gap, blocking the passing of blood contents (Rahimi et al., 2017). Besides, these two proteins also have a protective effect on endothelial cells against hazardous stimuli. For example, knockdown of occludin potentiated cytokine secretion, inflammasome activation, and pyroptosis occurrence in TNF-α–treated bEnd.3 cells (Zhang et al., 2020).

It should be noted that claudin has some subtypes; however, it is acknowledged that claudin-1 is the main type of that expressed in the brain blood vessels. Berndt et al. reported that claudin-1 was expressed in human brain capillaries as the strongest claudin, even significantly more than claudin-5 (Berndt et al., 2019). A recent study showed that claudin-1 replaced claudin-5 at the TJs of brain capillary endothelial cells during the regeneration phase after stroke (Sladojevic et al., 2019). Furthermore, knockout of claudin-1 in null mice is lethal, suggesting its irreplaceable role for TJ preservation (Furuse et al., 2002).

Cerebral ischemia induces a rapid degradation of claudin and occludin, which disrupts the TJ structure and renders the endothelial cells more vulnerable, accelerating their damage (Zhang et al., 2017). These finally result in the collapse of the blood vessel barrier and severe edema in the brain (Sandoval et al., 2008). In contrast, preventing the loss of claudin and occludin can alleviate brain edema and lesions (Takenaga et al., 2009; Stamatovic et al., 2016).

In the present study, the administrations of SLT significantly and dose dependently reduced the infarct size, alleviated neural morphological injuries, and improved neurological functions in rats 24 h after MCAO, indicating that SLT has an explicit protective effect against bran ischemia. Additionally, SLT treatments also significantly decreased the brain water content and elevated the expressions of endothelial TJ proteins including claudin-1 and occludin. This result is in accordance with our previous reports, and further hints that SLT may exert its therapeutic effects on brain ischemia through protection of cerebral vascular endothelial cells (Seto et al., 2017; Fan et al., 2020).

Cerebral microvascular endothelium is highly vulnerable to oxidative stress. It has been suggested that the disruptions of blood vascular endothelial TJs and endothelial cell damage in brain infarction are all closely related to peroxidation (Musch et al., 2006; Freeman et al., 2012).

Oxidative stress refers to a relative surplus of ROS, which is a sort of oxygen molecule derivatives having an extremely vivacious oxidative activity and mainly includes super oxygen anions, hydroxyl radicals, and hydrogen peroxides. The origin of ROS in endothelial cells includes the mitochondrial electronic transition chain, the cytoplasm hypoxanthine–xanthine oxidase system, and NAPDH oxidase system (Fraser et al., 2011). Usually, super oxygen anions are first generated, and are then soon metabolized into hydroxyl radicals and hydrogen peroxides, which even have more strong oxidative abilities.

The targets of ROS are mainly biological macromolecules including DNA in the nucleus and in the mitochondria, cytomembrane lipids, and proteins. ROS break their unsaturated links, leading to severe abnormalities in their structures and functions, which further cause disorders in cellular signal transductions and organelle functions; even generate cessation in pivotal physiological procedures such as energy metabolism, cellular structure repairs, and eliminations of damaged organelles; and finally result in cell degenerations, even cell death (Olmez et al., 2012).

The Nrf2 pathway is the major antioxidation system in most cell types, including vascular endothelial cells. Nrf2 is a nuclear transcription factor; however, in the quiescent state, most of which are detained in the cytoplasm and faced to ubiquitin-mediated degradation (Itoh et al., 1999). Upon oxidative assaults, Nrf2 is released, then translocates into the nucleus, and combines with DNA, promoting the transcriptions of a series of antioxidative genes including HO-1, SOD, and GST-1 (Otterbein et al., 2000; Harvey et al., 2009). HO-1 catalyzes the generation of carbon monoxide, which has strong reduction ability; SOD degrades super oxygen anions; and GST-1 promotes the generation of GSH, which is a powerful and broad-spectrum ROS scavenger. Thus, with these potent downstream effectors, the Nrf2 pathway exerts an efficient elimination on free radicals, making a mighty counterattack to the oxidation assault.

However, interestingly, this passive defense system can play active roles in some conditions. In the ischemia preconditioning, small amount ROS generated from a transient ischemia-reperfusion can activate the Nrf2 pathway, endowing the cells strong antioxidation ability to confront the following severe ischemia challenge (Nguyen et al., 2009; Zhang et al., 2017). Another example is the preconditioning-like effects elicited by some chemicals, which have strong redox activities and can liberate Nrf2 in the similar way with ROS (Ashabi et al., 2015; Ding et al., 2015). Many natural substances have this effect, such as some dietary phytochemicals including epicatechin, catechin, quercetin, and kaempferol; more importantly, numerous Chinese herbal ingredients are also included, among which the famous ones comprise salviolic acids, ligustrazine, and luteolin. Thus, the Nrf2 pathway forms a bridge for natural compounds to exert the physical modulatory effects (Alfieri et al., 2013; Hu et al., 2018; Li et al., 2018; Farkhondeh et al., 2019; Yamagata et al., 2020).

In consideration of the key role of peroxidation in vascular endothelium damage in ischemia and the key role of Nrf2 in antioxidation, we next investigated the role of the Nrf2 pathway in SLT’s endothelial protective effects. In *in vivo* experiments, SLT treatments significantly elevated the function of Nrf2 in the brain infarction; accordingly, the expression of HO-1, activity of SOD, and content of GSH in the brain were all increased by SLT, indicating the downstreams of Nrf2 were activated. Notably, the immunochemistry examination positioned this Nrf2 pathway activation to cerebral vascular endothelial cells. These data preliminarily demonstrate the central role of the Nrf2 pathway in SLT’s vascular endothelium protection.

To obtain the direct evidence, we performed an *in vitro* experiment. We selected hCMEC/D3, a human brain microvascular endothelial cell line as the experimental material. Interestingly, this cell line was found to express claudin-1 as one of the most dominant claudin subtypes, which is similar to the situation of *in vivo* brain vascular endothelial cells (Bederson et al., 1986; Brouns et al., 2009; Andjelkovic et al., 2019). And we selected the OGD/R model in which the cells are incubated with glucose-free medium in an anoxia circumstance to simulate ischemia *in vivo* and are then returned to normal medium and circumstance to simulate reperfusion.

**FIGURE 9:**
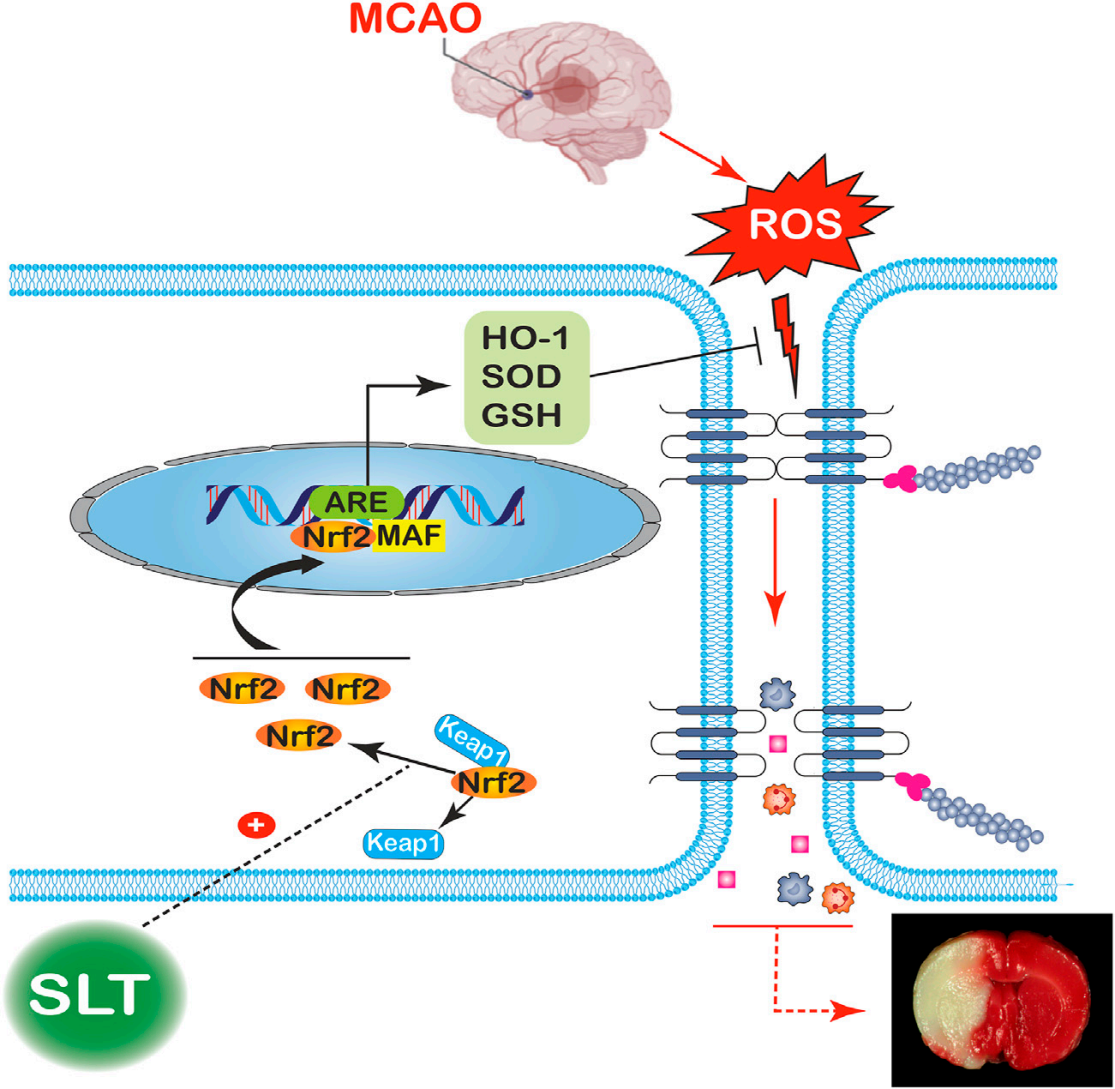
Putative mechanism for the therapeutic effect of SLT on brain ischemia. Occlusions in the cerebral arteries induce a drastic elevation in ROS contents; the excess ROS severely disrupts the brain blood barriers, leading to serious leakages from the blood vessels and brain edema, finally resulting in an infarction of large volume. SLT dramatically releases Nrf2 detained in the cytoplasm and promotes its nucleus translocation, thus priming the downstream transcriptions and increasing the contents of HO-1, SOD, and GSH, which then lead to massive elimination of ROS, cutting off the injury cascade and finally rescuing the brain from ischemia.

At first, SLT significantly inhibited OGD/R-induced drop in viabilities of hCMEC/D3 cells, confirming its direct protective effects on brain vascular endothelial cells against ischemia. Moreover, the Nrf2 pathway was also activated in hCMEC/D3 cells by SLT treatments, further revealing the relationship between Nrf2 pathway and SLT’s therapeutic effects. Last, we used siRNA to block the function of Nrf2, and expectedly, the protective effects of SLT on hCMEC/D3 cells against OGD/R injury were all diminished, conclusively confirming the obligatory role of the Nrf2 pathway in SLT’s protection on the brain vascular endothelium. Furthermore, combining the results of *in vivo* and *in vitro* experiments, it can be inferred that the Nrf2 pathway–dependent protection on cerebral microvascular endothelial cells underlies the therapeutic effect of SLT on brain ischemia (the putative mechanism of SLT’s anti-brain ischemia effect is shown in Figure 9).

However, as a redox active drug, SLT also has the ability to directly scavenge ROS, then does this activity play a role in SLT’s effects? As shown in our experiment results, the knockdown of Nrf2 expression resulted in a nearly total ablation in SLT’s protective effects on hCMEC/D3 cells; thus, it indicates that the direct antioxidation effect of SLT does not make a significant contribution to its vascular endothelium protection in brain ischemia.

At last, an interesting issue should be discussed. SLT has multiple ingredients, then which exert this Nrf2-dependent therapeutic effect? The main ingredients of SLT include ginsenosides, flavones, ginkgolides, and crocin. Although, according to the published studies, all of them have active redox characteristics and the potential to activate Nrf2, there exist differences in their activities. Flavones and crocin possessed the strongest redox activities, which are far more than that of the others (Ma et al., 2012; Barbagallo et al., 2013). Additionally, their amounts occupy nearly a half of that of SLT’s main active ingredients. Thus, the flavones and crocin in SLT seem to be associated most with the Nrf2-dependent endothelium protection. However, what are the exact roles of SLT’s ingredients in its therapy, and what are the relationships between them, simple additive relation, or synergic relation, or even antagonistic relation, need to be further studied in the future.

This study also has some limitations. In *in vitro* experiments, we used a monoculture of brain microvascular endothelial cells (BMECs). Although BMECs are a major and key component of the BBB, they are not the only one as astrocytes and pericytes also participate in the constitution of the BBB (Rahman NA et al., 2016). Thus, to further understand the effects of SLT on the BBB, an *in vitro* coculture model of BMECs with astrocytes or pericytes may be necessary. We consider performing related experiments in the future studies.

In conclusion, data of this study suggest that SLT’s prevention against brain ischemia is related to its protection on the cerebral vascular endothelial cells, which is dependent on the activation of the Nrf2 antioxidation pathway.

## Data Availability

The original contributions presented in the study are included in the article; further inquiries can be directed to the corresponding authors.
